# Diverse Patterns and Impact of Mitochondrial DNA Alteration in Parietal Cortex of Diabetic and Non‐diabetic Alzheimer’s Disease

**DOI:** 10.1002/alz.089513

**Published:** 2025-01-03

**Authors:** Anqi Shi, Aakruti Kaikini, Alan Hodgkinson, Afshan N Malik

**Affiliations:** ^1^ King’s College London, London, London United Kingdom

## Abstract

**Background:**

Diabetes increases the risk of Alzheimer’s disease (AD), and mitochondrial dysfunction is implicated in both diseases. We previously detected mitochondrial DNA copy number (MtDNA‐CN) changes in human parietal cortex that differed between diabetic AD and non‐diabetic AD. We hypothesize that MtDNA‐CN changes may be indicative of different underlying mechanisms. In the current study, we aim to evaluate the impact of MtDNA‐CN changes on MtDNA damage and proteins involved in energy production in the mitochondria.

**Method:**

Total DNA and protein were isolated from human post‐mortem parietal cortex (n = 24). MtDNA‐CN was determined as the ratio of MtDNA to nuclear DNA using qPCR. MtDNA damage was measured using the surveyor nuclease method in 11 overlapping mitochondrial genome regions, and next generation sequencing (NGS) after removing the nuclear DNA using exonuclease V.

NDUFB8(I), SDHB(II), UQCRC2(III), MTCO1(IV), and ATP5A(V) expression were measured as the representatives of 5 complexes in OXPHOS chain by western blotting. Data were compared as 4 groups: non‐cognitive impairment (NCI) and AD, ± diabetes using ANOVA.

**Result:**

In AD groups, MtDNA‐CN was reduced in cases without diabetes (P<0.05), however, no reduction was seen in diabetic AD. In diabetic groups, irrespective of cognitive status, higher MtDNA‐CN were found compared to non‐diabetic groups. Putative mutations with various intensities and patterns were observed, especially in the displacement loop area in AD and diabetic AD groups. Among 4 groups, the expression of 2 nuclear‐encoded subunits NDUFB8 and ATP5A were higher than the level of other 2 nuclear‐encoded subunits (SDHB and UQCRC2) and 1 mitochondrial‐encoded subunit MTCO1. However, no significant difference was found in the expression of these 5 subunits between diabetic and non‐diabetic groups in parietal cortex.

**Conclusion:**

Our data show that in human parietal cortex, the MtDNA‐CN changes differently in diabetic and non‐diabetic AD suggesting of different underlying mechanisms. The presence of MtDNA changes without any impact on levels of OXPHOS subunits indicates an MtDNA‐based pathology. We propose that different patterns of MtDNA damage may impact energy production in specific brain regions and contribute to disease progression. The level of MtDNA heteroplasmy in the groups determined by NGS and its potential impact will be presented.

